# Myeloid/lymphoid neoplasm with eosinophilia and rearrangement of PDGFRA presenting as myeloid sarcoma

**DOI:** 10.1002/jha2.477

**Published:** 2022-06-08

**Authors:** Mohammad Barouqa, Dragan Jevremovic, Patricia Theresa Greipp, James Hoyer, Kaaren Reichard, Ellen D. McPhail

**Affiliations:** ^1^ Mayo Clinic Rochester Minnesota USA

1

A 32‐year‐old male patient with no significant past medical history presented with fatigue and left groin lymphadenopathy. The left groin lymph node was excised and measured 2.0 × 1.4 × 1.1 cm. On histologic examination, the lymph node architecture was effaced by atypical cells with intermediate to large nuclei, round nuclear contours, vesicular chromatin, prominent nucleoli, and moderate basophilic cytoplasm admixed with abundant eosinophils (Figure 1, top left, objective 40×). By immunohistochemistry, the neoplastic cells express CD13 (subset), CD33 (weak), CD34 (weak), CD45, CD68/PG‐M1 (subset), CD71 (top right, objective 40×), CD117 (subset), CD123 (weak), and Hemoglobin A (subset). Given the extensive eosinophilic infiltration, FISH analysis for PDGFRA (4q12) and PDGFRB (5q33) was performed. A rearrangement of the *PDGFRA* (4q12) gene region with loss of 5′ *PDGFRA* and retention of 3′ oncogenic portion was identified in 63% of nuclei (Figure 1. bottom left, *PDGFRA* 5′(G) / 3′(R) [4q12], arrows indicate disruption of PDGFRA). No abnormality of *PDGFRB* was observed. FISH analyses for the *ABL1*, *BCR*, *MYH11*, and *CBFB* gene regions were within normal limits. Because of these findings, a peripheral blood smear, and bone marrow aspiration and biopsy were performed. The patient's blood smear demonstrated marked eosinophilia (59% of the white blood cell count differential) but no blasts. The bone marrow aspirate was hemodilute. The bone marrow core biopsy contained rare, atypical cells that were morphologically like those present in the lymph node, accompanied by numerous mature eosinophils (Figure 1, bottom right, objective 40×) and a small population of mast cells with spindled morphology. The atypical cells expressed the same antigens as their counterparts in the lymph node, and mast cells coexpressed CD25, CD117, tryptase but were negative for CD2. FISH performed on the peripheral blood showed *a PDGFRA* (4q12) gene rearrangement in 5% of nuclei. All other FISH studies were within normal limits (11q23 (*MLL*(*KMT2A*) sep), t(8;21) *RUNX1T1::RUNX1* fusion, t(15;17) *PML::RARA* fusion, inv(16)/t(16;16) *MYH11::CBFB* fusion, ‐5q31(D5S630 × 2,EGR1 × 1), ‐5(D5S630,EGR1)x1,‐7q31(D7Z1 × 2,D7S486 × 1), ‐7(D7Z1,D7S486)x1, inv(3) *RPN1::MECOM* fusion, t(6;9) *DEK::NUP214* fusion, ‐17p13.1(TP53 × 1,D17Z1 × 2), ‐17(TP53,D17Z1)x1, and(9;22) *ABL1::BCR* fusion), and molecular analyses for *KIT* Asp816Val, *JAK2* V617F, *CALR* exon 9, *MPL* exon 10 were negative for mutations. Next generation sequencing (NGS) for myeloid neoplasms revealed a variant of unknown significance (*SH2B3*: Chr12(GRCh37): g.111886029C > T; NM_005475.2(SH2B3): c.1651C > T; p.Arg551Trp (49%)).

**FIGURE 1:**
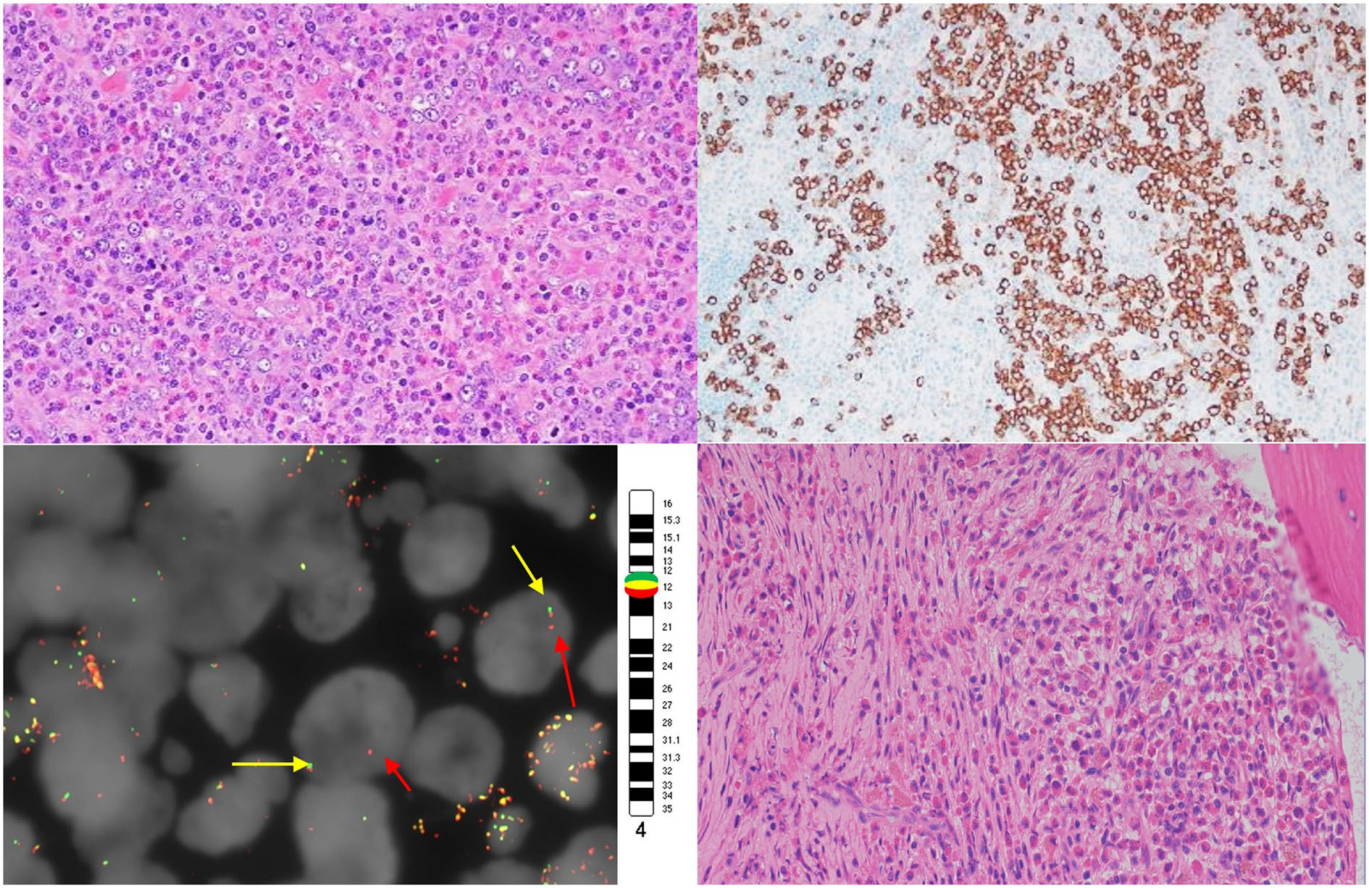
Top left, H and E stain performed on section from lymph node. Top right, CD71 stain performed on section from lymph node. Bottom left, arrows indicate disruption of PDGFRA. Bottom right, H and E stain performed on bone marrow core biopsy

“Myeloid/lymphoid neoplasm with eosinophilia (M/LNs‐Eo) and rearrangement of *PDGFRA*, *PDGFRB or FGFR1*, or with *PCM1‐JAK2*” is recognized as a specific diagnostic category in the 2017 World Health Organization (WHO) classification. Recognition of these neoplasms is crucial given that genetic rearrangements of *PDGFR1* and *PDGFRB* render them exquisitely sensitive to tyrosine kinase inhibitors. We present an unusual case of a PDGFRA‐rearranged myeloid sarcoma with associated bone marrow involvement by a *PDGFRA*‐rearranged chronic myeloid neoplasm. Spindle‐shaped, CD25‐positive mast cells are common in *PDGFRA*‐rearranged chronic myeloid neoplasms of the bone marrow and are not considered to be indicative of systemic mastocytosis. The presence of eosinophils in both medullary and extramedullary sites should prompt testing for *PDGFRA*, *PDGFRB*, *FGFR1*, and *PCM1‐ JAK2* rearrangements, in light of the potential therapeutic implications.

## CONFLICT OF INTEREST

The authors declare they have no conflicts of interest.

## ETHICS STATEMENT

At Mayo Clinic single participant case studies, or a case series with multiple participants that are prepared and disseminated for educational purposes, are not systematic investigations and, therefore, are not considered research.

## AUTHOR CONTRIBUTIONS

Mohammad Barouqa wrote the manuscript and took images. Dragan Jevremovic reviewed the lymph node and edited the manuscript. James Hoyer reviewed the bone marrow and took images. Patricia Theresa Greipp reviewed the cytogenetics and edited the manuscript. Kaaren Reichard reviewed the bone marrow and edited the NGS on the manuscript. Ellen McPhail supervised and edited the manuscript.

